# Balancing Thrombosis and Bleeding: Antithrombotic Therapy in Cirrhosis-Related Thrombocytopenia

**DOI:** 10.3390/jcm15052036

**Published:** 2026-03-07

**Authors:** Sarah Taylor, Julie Wang, Hui Yin Lim, Glen Saward, Siddharth Sood

**Affiliations:** 1 Department of Gastroenterology, Northern Health, Epping, VIC 3076, Australia; sarah.taylor12@nh.org.au (S.T.); glen.saward@nh.org.au (G.S.); 2 Northern Clinical Diagnostics & Thrombovascular Research (NECTAR), Northern Health, Epping, VIC 3076, Australia; julie.wang@nh.org.au (J.W.); huiyin.lim@nh.org.au (H.Y.L.); 3 Department of Medicine, The University of Melbourne, Parkville, VIC 3010, Australia; 4 Department of Haematology, Northern Pathology Victoria, Northern Health, Epping, VIC 3076, Australia; 5 Australian Centre for Blood Diseases, Monash University, South Yarra, VIC 3181, Australia; 6 School of Health and Biomedical Sciences, RMIT University, Melbourne, VIC 3001, Australia

**Keywords:** cirrhosis, thrombosis, thrombocytopenia, anticoagulation

## Abstract

The development of thrombocytopenia is common in cirrhosis. Further complex alterations in haemostasis also occur, resulting in a rebalanced state that predisposes patients to both thromboembolic and bleeding complications. Guidance on the management of thrombosis in patients with cirrhosis-related thrombocytopenia is limited and poses a common clinical dilemma. Anticoagulation in this population remains challenging due to altered drug pharmacokinetics, baseline abnormalities in conventional coagulation tests, limitations in laboratory monitoring, thrombocytopenia itself and concerns regarding bleeding risk. Low-molecular-weight heparin and vitamin K antagonists have traditionally been used; however, increasing data support the use of direct oral anticoagulants in patients with compensated cirrhosis. Management decisions should be individualised, incorporating liver disease severity, thrombotic burden, bleeding risk, and clinical factors such as portal hypertension. This review summarises current evidence on thromboembolic disease and antithrombotic therapy in cirrhosis-related thrombocytopenia. Further prospective studies are required to investigate key knowledge gaps, including optimal platelet thresholds for anticoagulation use and the role of functional coagulation testing in this population.

## 1. Introduction

Cirrhosis constitutes a significant global health burden, responsible for approximately 2.4% of deaths worldwide in 2019 [1]. Chronic viral hepatitis B and C have been the predominant causes, but effective therapies are reducing their incidence and improving outcomes. In contrast, the incidence of metabolic dysfunction-associated steatotic liver disease (MASLD) is increasing [1]. Disease severity in cirrhosis is commonly classified using the Child–Pugh score (Table 1), with higher scores associated with increased risk of hepatic decompensation and mortality [2,3]. Increasing hepatic fibrosis contributes to increased vascular resistance that often results in portal hypertension. Development of portal hypertension is frequently associated with complications, including variceal bleeding and ascites, along with thrombocytopenia [4].

Thrombocytopenia is typically defined as a platelet count of <150 × 10^9^/L, with moderate thrombocytopenia between 50 and 100 × 10^9^/L and severe being <50 × 10^9^/L [5]. Approximately 70% of patients with cirrhosis have chronic thrombocytopenia, with 1–8% having severe thrombocytopenia [6,7]. Thrombocytopenia itself is an independent predictor of advanced liver disease and poorer prognosis [8]. Paradoxically, despite thrombocytopenia and perceived bleeding risk, cirrhosis is often associated with increased risk of thromboembolism [6,9]. Portal venous thrombosis is a well-recognised complication in cirrhosis, with a 5-year cumulative incidence of 10.7% reported in a large multicentre randomised controlled trial [10]. Rates of deep venous thrombosis (DVT), pulmonary embolism (PE) and arterial thromboembolism have also been shown to be more frequent in patients with cirrhosis [11].

This dual risk reflects the concept of rebalanced haemostasis in cirrhosis, a fragile equilibrium resulting from impaired hepatic synthesis of both procoagulant and anticoagulant factors. In addition, haemostasis is further altered through a combination of thrombocytopenia, increased circulating von Willebrand factor (vWF), and reduced activity of ADAMTS13 (a disintegrin and metalloproteinase with thrombospondin type 1 motifs, member 13). Finally, both qualitative and quantitative abnormalities in fibrinolytic pathways may contribute to dysregulated fibrinolysis in patients with cirrhosis [12,13,14]. This rebalanced state can be easily disrupted by insults, such as infection, leading to a shift towards either thrombosis or haemorrhage [15,16].

With the growing burden of cirrhosis, clinicians increasingly encounter scenarios where antithrombotic therapy must be considered. Traditional options include low-molecular-weight heparin (LMWH) and vitamin K antagonists, but emerging evidence supports the cautious use of direct oral anticoagulants (DOACs) in selected patients. Although coexistent thrombocytopenia is commonly encountered in this population, most management guidelines provide inadequate guidance on anticoagulation in patients with associated thrombocytopenia. Optimal management remains challenging and requires individualised strategies informed by evolving data.

This review aims to examine the complex interplay between thrombosis and bleeding in cirrhosis-related thrombocytopenia, evaluate current evidence on antithrombotic strategies and provide practical considerations for individualised management in this high-risk and challenging scenario.

## 2. Pathophysiology of Thrombocytopenia in Cirrhosis

Thrombocytopenia is more common in those with advanced liver disease (Child Pugh C), though those with compensated cirrhosis (Child Pugh A) may still have severe thrombocytopenia [17]. Thrombocytopenia in cirrhosis is multifactorial, arising from a combination of decreased platelet production, increased platelet destruction, and splenic sequestration [18] (Table 2).

Reduced platelet production is driven by impaired thrombopoietin (TPO) synthesis, a key regulator of platelet formation, and bone marrow suppression. TPO is predominantly synthesised in the liver and acts by binding to the c-MPL receptor on megakaryocytes and haematopoietic stem cells. This activates several intracellular pathways, leading to the differentiation of bone marrow stem cells into megakaryocytes and the production of platelets. Furthermore, c-MPL expression itself may be downregulated, compounding the defect in platelet production. Bone marrow suppression from alcohol use, chronic hepatitis C infection and iron overload in haemochromatosis-related cirrhosis may also occur [18].

Increased platelet destruction contributes to thrombocytopenia through several mechanisms, including reduced levels and activity of ADAMTS13, immune-mediated platelet destruction, and inflammation associated with bacterial infections. ADAMTS13, produced by hepatic stellate cells, cleaves von Willebrand factor (vWF). Subsequent decreases in ADAMTS13 activity lead to increased vWF multimers and heightened platelet aggregation. Immune-mediated thrombocytopenia may occur in patients with autoimmune liver disease and chronic hepatitis C infection. Additionally, cirrhosis increases susceptibility to infections, which may cause consumptive thrombocytopenia through systemic inflammation and activation of coagulation pathways [19,20].

Finally, splenic sequestration is a common consequence of portal hypertension. Increased splenic venous blood flow and congestion result in pooling of platelets within the enlarged spleen, reducing circulating platelet counts [18]. However, severe thrombocytopenia (platelet count of <25 × 10^9^/L) is rarely attributable to portal hypertension alone, and should prompt consideration of alternative aetiologies such as immune thrombocytopenia [21].

## 3. Thrombosis in Cirrhosis

Cirrhosis is characterised by a complex rebalancing of haemostatic pathways, with accumulating evidence suggesting that this altered state may favour thrombosis rather than bleeding [13]. This prothrombotic tendency arises from multiple mechanisms, including alterations in pro- and anticoagulant factors, as well as systemic contributors such as venous stasis and chronic inflammation. Collectively, these changes are associated with approximately a 2-fold increased risk of venous thromboembolism compared with the general population [9]. Malnutrition, black population, hypoalbuminemia and central venous lines have also been associated with increased rates of venous thrombosis in cirrhosis [22,23,24].

Patients with more advanced or decompensated liver disease appear to be at the most significant risk of thromboembolic complications, in particular portal venous thrombosis (PVT) [25]. Several studies have reported PVT rates ranging from 0.6% to 15% in cirrhosis, increasing to as high as 35% in patients with end-stage disease [26]. PVT is thought not only to occur due to altered haemostatic factors but also to be largely influenced by altered portal haemodynamics, with overall reduced portal flow. In cases where anticoagulation fails to achieve recanalisation, interventions such as a transjugular intrahepatic portosystemic shunt (TIPS) or chemical/mechanical thrombectomy may be considered, as restoration of the portal vein flow can improve outcomes [27,28].

Careful decision-making around anticoagulation is required in patients with cirrhosis and PVT. This includes balancing factors such as the potential for future liver transplantation, chronicity, extent of thrombus, including superior mesenteric vein (SMV) involvement, and bleeding risks. There is an increasing tendency to anticoagulate patients with acute (<6-month) portal vein thrombosis involving greater than 50% occlusion of the main portal vein or mesenteric veins, given the associated risks of progression and adverse clinical outcomes [27,29]. The decision to anticoagulate a patient with a chronic PVT is more nuanced and often only recommended in those with additional thrombosis risk factors, mesenteric involvement, bowel ischemia or in patients being considered for liver transplantation. This is based on limited retrospective data [30].

Patients who develop PVT without a prior history of thrombosis generally do not require further thrombophilia investigation. However, further evaluation may be warranted in those with recurrent events or a family history of thrombosis. Interpretation of thrombophilia assays in cirrhosis is challenging, as the disease itself reduces levels of protein C, S and antithrombin, complicating differentiation between inherited and acquired deficiencies [27,29].

Systemic venous thromboembolism (VTE), including deep vein thrombosis and pulmonary embolism, occurs at approximately twice the rate observed in patients without cirrhosis, affecting up to 6% of individuals [31]. Large cohort studies have demonstrated that MASLD is associated with a higher risk of venous thromboembolism compared with alcohol-related liver disease and viral hepatitis [32]. Given that rates of MASLD cirrhosis are increasing worldwide, VTE is likely to become an increasingly common complication in this population.

Arterial thrombotic events are also more common in cirrhosis. In a large Danish cohort study, ischemic stroke risk was elevated in those with cirrhosis (adjusted hazard ratio (HR) 1.7, 95% CI 1.3–2.3), with higher rates of myocardial infarction reported in those with decompensated compared to compensated cirrhosis (adjusted HR 8.7, 95% CI 2.7–28.3). Both arterial thromboembolic conditions were associated with increased mortality in those with cirrhosis in comparison to those without liver disease [11]. Unlike portal venous thrombosis, the severity of liver disease has not been associated with an increased risk of PE/DVT [24].

## 4. Assessment of Bleeding and Thrombosis Risk with Cirrhosis-Related Thrombocytopenia

Thrombocytopenia and thrombosis commonly co-exist in cirrhosis, and careful individualised assessment is required to balance the competing risks of bleeding and thrombosis before the initiation of anticoagulation therapy. Regular re-evaluation during anticoagulation therapy should also occur. Whilst thrombocytopenia is not an absolute contraindication to anticoagulation in cirrhosis, it is an important consideration that may influence the intensity and duration of anticoagulation [27]. However, as discussed in subsequent sections of this review, the quality of evidence to inform clinical decision-making is largely based on extrapolated data from non-cirrhotic cohorts and expert consensus.

Factors that may increase bleeding risk in liver disease should be optimised in those who develop thrombosis requiring anticoagulation. This includes, where feasible, reducing portal pressure with non-selective beta-blockers. Endoscopic assessment and variceal eradication should also be performed, though guidelines regarding the timing relative to the commencement of anticoagulation vary. Improved outcomes are observed when anticoagulation is initiated within 2 weeks of an acute thrombosis [33]. In the setting of concurrent significant cirrhosis-related thrombocytopenia, the authors recommend performing screening endoscopy prior to initiating anticoagulation. This is based on limited evidence and requires further research [27].

### 4.1. Global Coagulation Assays to Assess Bleeding Risk

Traditional coagulation tests provide an unreliable assessment of bleeding risk in patients with cirrhosis. Prothrombin time (PT) and activated partial thromboplastin time (APTT) only measure time to clot formation and do not reflect the reduction in natural anticoagulants in cirrhosis, or the rebalanced haemostatic state. Global coagulation assays, such as Thrombin Generation (TG) assays with thrombomodulin, better reflect the rebalanced coagulation state in cirrhosis. TG measures both pro- and anticoagulation factors through thrombin formation and inhibition. The addition of thrombomodulin in this assay normalises thrombin generation, indicating that decreased anticoagulant factors counterbalance the deficits in thrombin generation in cirrhosis. Clinical application of TG in cirrhosis, however, has failed to accurately predict bleeding or thrombosis. Additionally, TG assays are typically performed in platelet-poor plasma, which does not accurately reflect in vivo haemostasis conditions [34].

A prospective study of 230 patients with compensated and decompensated cirrhosis evaluated bleeding, thrombosis, hepatic decompensation, and liver-related mortality. All participants underwent both whole-blood (WB) and platelet-poor plasma (PPP) thrombomodulin-modified thrombin generation (TG) assays, with a median follow-up of 414 days [34]. In patients with compensated cirrhosis, neither WB-TG nor PPP-TG predicted bleeding outcomes. In contrast, among those with decompensated cirrhosis, WB-TG demonstrated a strong predictive ability for periprocedural major bleeding (area under the curve, 0.854; 95% CI: 0.732–0.976; *p* < 0.001). Furthermore, a WB-TG endogenous thrombin potential >700 nmol/L min in decompensated cirrhosis was independently associated with a reduced risk of further hepatic decompensation, acute-on-chronic liver failure, and liver-related mortality (HR 0.40, 95% CI: 0.21–0.79; *p* < 0.01). Neither WB-TG nor PPP-TG was predictive of thrombotic events. These findings suggest a potential role for WB-TG in risk stratification for bleeding and clinical outcomes in decompensated cirrhosis; however, further studies are required to better define the utility of TG assays in predicting bleeding risk in patients with cirrhosis-related thrombocytopenia [35].

Viscoelastic testing (VET), including ROTEM (ROtational ThromboElastoMetry) and TEG (ThromboElastoGraphy), offers a more comprehensive assessment of coagulation and is primarily used to guide blood product replacement during invasive procedures. Randomised controlled trials in cirrhosis have demonstrated that VET can reduce blood product requirements during invasive procedures [36,37]. The ability of VET to predict thrombosis is limited, possibly because it is insensitive to vWF and protein C, which are altered in cirrhosis [38]. A prospective cohort study of 162 patients with cirrhosis, followed up for 1 year, found no association between ROTEM parameters and the risk of thrombosis [39]. Further studies are required to assess the role of VET in thrombosis prediction [39].

### 4.2. Platelet Function Testing to Assess Bleeding Risk

Studies of platelet function have yielded inconsistent results with reports of both increased and decreased platelet aggregation. One study of 34 patients with cirrhosis assessed in vitro platelet function using light transmission aggregometry, measurement of platelet granules, flow cytometry and thrombin generation in platelet-rich plasma [40]. Thrombin generation measured in platelet-rich plasma was similar between patients with cirrhosis and controls. However, platelets from cirrhotic patients demonstrated reduced aggregation and ATP secretion, along with diminished activation markers such as P-selectin expression and PAC-1 binding. Interestingly, plasma levels of β-thromboglobulin and soluble P-selectin were elevated in patients, indicating in vivo platelet activation, while platelet factor-4 levels were comparable between groups. These findings suggest a paradox: despite impaired in vitro aggregation, cirrhotic patients exhibit evidence of in vivo platelet activation, which may help maintain haemostatic potential during invasive procedures [40]. This aligns with clinical observations that bleeding events post-procedure are uncommon and supports international guideline recommendations against routine prophylactic platelet transfusion based solely on laboratory platelet function testing. Decisions regarding platelet support should be individualised and not driven by aggregometry results. These differences likely reflect methodological differences, cohort heterogeneity and the confounding effect of thrombocytopenia itself. Traditional diagnostic tools, such as platelet aggregometry, have poor reproducibility and limited predictive value for bleeding risk in cirrhosis, and increasing evidence argues against their use. This highlights the need for newer techniques to better predict bleeding risk in this population [40,41].

## 5. Transfusion Strategies and Thrombopoietic Therapies for the Management of Thrombocytopenia

### 5.1. Platelet Transfusions

Several studies have examined cirrhosis-related thrombocytopenia and its association with bleeding risk, including both spontaneous and peri-procedural bleeding. In a prospective cohort study of nearly 300 patients with cirrhosis followed for three years, no association was observed between absolute platelet count and the risk of spontaneous major or minor bleeding (HR 0.99, 95% CI: 0.995–1.004). Similarly, a platelet count below 50 × 10^9^/L was not associated with an increased risk of spontaneous bleeding (HR 0.65, 95% CI: 0.31–3.36) [6]. Given the limited evidence, routine platelet transfusion is not recommended for the prevention of spontaneous bleeding in patients with cirrhosis-associated thrombocytopenia [42].

Studies examining platelet count and procedural bleeding are more heterogeneous but overall suggest there may be an increased risk for those with severe thrombocytopenia undergoing high-risk procedures [43]. This, however, may be more reflective of underlying liver disease severity and portal hypertension than the platelet count itself [44]. In contrast, a meta-analysis including nearly 5000 patients with cirrhosis undergoing low-risk surgical procedures found no difference in bleeding rates between those with platelet counts below 50 × 10^9^/L and those with platelet counts above this threshold [45].

Guidelines regarding the use of platelet transfusion in patients with severe thrombocytopenia prior to invasive procedures remain inconsistent. Furthermore, the lack of clear and uniform definitions distinguishing high- from low-risk procedures further complicates the development and implementation of uniform guidelines. Both the British Society of Gastroenterology (BSG) and American College of Gastroenterology (ACG) recommend either platelet transfusion or thrombopoietin receptor agonists if platelets are below 50  × 10^9^/L [30,46]. The American Association for the Study of Liver Diseases (AASLD) does not routinely recommend platelet transfusion, and the American Gastroenterological Association (AGA) suggests discussing with haematologists [29,47]. The International Society on Thrombosis and Haemostasis (ISTH) and the European Association for the Study of the Liver (EASL) recommend considering transfusion based on the risk of surgery, the absolute platelet count, and the ability to achieve haemostasis [42,48,49].

In summary, prophylactic platelet transfusions are not recommended if the platelet count is >50 × 10^9^/L in both hepatology and haematology guidelines [44]. If bleeding does occur, a platelet count of >50 × 10^9^/L is generally targeted [44].

### 5.2. Thrombopoietic Therapies

Traditionally, platelet infusions have been used to increase platelet counts; however, their effect is short-lived, typically lasting 2.4–4.5 days, and they exhibit reduced functional capacity [50]. In recent years, the use of thrombopoietin receptor agonists (TPO-RAs) for cirrhosis-related thrombocytopenia has increased [50]. Most commonly, these have been used in the peri-procedural setting for patients with severe thrombocytopenia undergoing high-risk interventions such as central nervous system procedures [44]. These agents offer a more sustained increase in platelet counts without increasing portal pressure, making them an attractive alternative to transfusion [50].

However, there have been some concerns that treatment with TPO-RAs is associated with an increased risk of thrombosis in higher doses. A recent meta-analysis of 3 randomised controlled trials involving over 1900 patients with cirrhosis receiving TPO-RAs (eltrombopag, avatrombopag, and lusutrombopag) examined PVT rates. There was a trend toward a higher PVT rate in those treated with TPO-RAs, though this did not reach statistical significance (odds ratio (OR): 2.8; 95% CI: 0.97–8.16; *p* = 0.055). However, patients treated with eltrombopag had higher rates of PVT (OR: 3.8; 95% CI: 1.14–13.2; *p* = 0.03), as well as arterial and venous thrombosis. This may be due to higher doses of eltrombopag, resulting in higher platelet counts than with other TPO-RAs, approaching 200 × 10^9^/L [51].

## 6. Management of Concurrent Thrombosis and Thrombocytopenia in Patients Without Cirrhosis

Given limited cirrhosis-specific data, it is important to consider other disease states to guide thrombosis management in this setting. Co-existing thrombocytopenia and thrombosis can be observed in patients with malignancies (both solid organ and haematological), immune-mediated thrombocytopenia (ITP) and in the setting of sepsis [48].

In oncology and haematology, thrombocytopenia is frequently a consequence of cytotoxic chemotherapy and is routinely graded according to the Common Terminology Criteria for Adverse Events (CTCAE) (Table 3). This grading framework has been widely adopted in clinical trials and practice, providing a structured approach to balancing thrombotic and bleeding risks when considering anticoagulation in thrombocytopenic patients [52].

Guidelines, including those from the International Society on Thrombosis and Haemostasis (ISTH), have traditionally urged caution with anticoagulation in patients with platelet counts <50 × 10^9^/L and avoidance if <25 × 10^9^/L. They suggest reduced-dose low molecular weight heparin (LMWH) to a platelet count of 10 × 10^9^/L in the setting of very high-risk thrombosis. Most of this data is derived from cancer-associated thrombosis (CAT) and is primarily based on expert opinion from retrospective data [48]. These guidelines recommend basing anticoagulation decisions on the acuity and risk features of the thromboembolism, as well as the platelet count [48].

The management of patients with thrombosis and a platelet count <50 × 10^9^/L varies widely. One suggested strategy includes commencing full-dose LMWH with platelet transfusion support with a general platelet target of 40–50 × 10^9^/L, though the optimal platelet level has not been assessed [48]. Alternative approaches include reduced or prophylactic dose LMWH. There are no randomised controlled trials assessing these differing approaches, and a recent systematic review demonstrated no difference between them [48,53]. Thrombocytopenia in CAT is often a more transient condition, whereas the chronic nature of thrombocytopenia in cirrhosis makes it impractical for ongoing platelet transfusion support [48].

The majority of evidence for thrombosis treatment in CAT and thrombocytopenia is based on LMWH or unfractionated heparin (UFH) use in patients with renal failure [53]. There is also increasing evidence for DOACS. Randomised controlled trials assessing DOACS in patients with cancer have generally excluded individuals with platelet counts <100 × 10^9^/L or have not reported outcomes during periods of thrombocytopenia. Despite this, DOACs are increasingly used in this setting, largely due to their ease of administration and patient convenience [54].

A multicentre prospective cohort study of 120 patients with CAT and thrombocytopenia included 16 patients on DOACs. Of these, 16 patients were initiated on a DOAC at baseline (13 apixaban and 3 rivaroxaban), with a further 27 patients commenced on a DOAC during follow-up. The median platelet count was 51 × 10^9^/L (range 12–98). Over a median DOAC exposure of 62 days, three haemorrhagic events were observed, all of which were classified as clinically relevant non-major bleeding. No recurrent venous thromboembolic events occurred during the observation period [55]. A further study showed increased rates of bleeding in patients treated with rivaroxaban or edoxaban in comparison to LMWH in some subtypes of malignancy [48,56,57].

However, caution must be taken when extrapolating these data to patients with cirrhosis, as the underlying pathophysiology of the thrombocytopenia and thrombosis differs [49]. Additionally, the haemostatic changes in cancer vary markedly from those in cirrhosis, meaning direct comparison of bleeding and thrombosis risk between thrombocytopenic CAT and patients with cirrhosis may not be possible.

## 7. Antithrombotic Therapies in Cirrhosis

The management of thrombosis in cirrhosis is uniquely complex, requiring careful navigation of competing risks of bleeding and thrombosis within a fragile haemostatic balance. While anticoagulation can be safe and effective in selected patients, therapeutic decisions are complicated by pharmacological challenges inherent to liver disease. These include altered drug metabolism due to impaired hepatic function, variable renal clearance, and changes in plasma protein binding, all of which affect drug pharmacokinetics and pharmacodynamics. Additionally, portal hypertension and variceal bleeding risk necessitate individualised strategies that differ from standard approaches in non-cirrhotic populations. This section reviews available antithrombotic options alongside emerging evidence and practical considerations for optimising therapy in this high-risk group (Table 4).

### 7.1. Unfractionated Heparin/Low Molecular Weight Heparin and Vitamin K Antagonists

Multiple studies support the use of unfractionated heparin and low molecular weight heparin in patients with thromboembolism, but data supporting their use in patients with cirrhosis are more limited. Unfractionated heparin and LMWH activate antithrombin, which accelerates the inactivation of coagulation enzymes thrombin (factor IIA), factor Xa and factor IXA [58]. Warfarin is the most widely used vitamin K antagonist (VKA), which inhibits vitamin K-dependent coagulation factors [59]. These medications are appealing in cirrhosis due to their ease of monitoring, clinician familiarity and reversibility. Multiple guidelines recommend LMWH, particularly in patients with advanced liver disease (Child-Pugh B and C). UFH is recommended in patients with concomitant renal failure [42,47].

LMWH and UFH use in cirrhosis is complicated by the associated haemostatic changes that occur in this population. Increased response to LMWH has been demonstrated in patients with cirrhosis despite lower measured anti-thrombin and anti-Xa levels, leading to potential overdosing [60]. Additionally, monitoring these patients can be difficult due to lower anti-Xa levels associated with lower antithrombin levels [61]. UFH monitoring is further complicated in cirrhosis due to an often elevated baseline activated partial thromboplastin time [48].

Before the advent of novel anticoagulants, warfarin was considered the standard therapeutic choice for thromboembolism in the setting of both compensated and decompensated cirrhosis, although evidence was limited to observational studies and retrospective case series [44]. Its use can be limited by a baseline elevation in international normalised ratio (INR) that poses difficulty in setting and treating to a therapeutic target [62]. No studies to date have addressed the suitability of an INR target of 2–3 in patients with cirrhosis [42].

### 7.2. Direct Oral Anticoagulants

Although initially avoided due to concerns about variable metabolism, inability to reverse and difficulty in monitoring, DOACS are being increasingly used in patients with cirrhosis [42]. Hepatology guidelines support their use in compensated cirrhosis [29,37]. Their appeal lies in the ease of administration, fixed dosing and avoidance of routine laboratory monitoring.

Direct oral anticoagulants (DOACs) are classified into Factor Xa inhibitors (including rivaroxaban, apixaban, and edoxaban) and direct thrombin inhibitors (including dabigatran) [63]. Hepatic metabolism plays a significant role in their clearance, with approximately 75% for apixaban, 65% for rivaroxaban, 50% for edoxaban, and 20% for dabigatran [64]. Consequently, impaired liver function can lead to elevated plasma levels and increased bleeding risk.

Apixaban has been shown to have a similar area under the concentration-time curve (AUC) in patients with cirrhosis compared with healthy individuals and is thus suitable for use in both Child-Pugh A and B cirrhosis. Rivaroxaban has a 2.27-fold increase in AUC in Child-Pugh B cirrhosis. Twice daily dosing for apixaban vs. daily dosing for rivaroxaban may also contribute to bleeding risk with fluctuating plasma concentrations [65]. Rivaroxaban is therefore generally not recommended in Child-Pugh B cirrhosis, though there is increasing evidence that there may not be increased rates of major bleeding with its use [66]. Dabigatran is recommended with caution in Child-Pugh B cirrhosis due to its reliance on renal clearance, long half-life and more limited data [67]. A reduced dose of apixaban and dabigatran can be considered in those with Child-Pugh B cirrhosis [42]. These pharmacokinetic considerations highlight the importance of assessing liver disease severity when selecting and dosing DOACs in patients with cirrhosis.

Several retrospective and more recently prospective studies have evaluated the use of DOACS in cirrhosis. Most of these studies have combined DOACS use in the setting of both atrial fibrillation (AF) and venous thrombosis. Patients with severe liver disease (Child Pugh C), who are more likely to have significant portal hypertension and worse thrombocytopenia, have commonly been excluded from studies evaluating treatment of thromboembolism [68]. A recent large retrospective cohort study of over 16,000 patients with Child–Pugh C cirrhosis and atrial fibrillation compared outcomes in patients receiving anticoagulation, including 20.2% treated with DOACS, with those who were not anticoagulated. Using 1:1 propensity score matching, anticoagulation was associated with a significant mortality benefit at three years (40% vs. 72%, *p* < 0.0001) and a longer median survival (898 vs. 65 days), as well as a lower risk of embolic stroke. There was no significant difference in gastrointestinal haemorrhage between anticoagulated and non-anticoagulated patients (18.8% vs. 19.5%, *p* = 0.3); however, intracranial haemorrhage occurred more frequently in those receiving anticoagulation (6.2% vs. 4.9%, *p* = 0.03). Those on DOACS (vs warfarin) had lower rates of intracranial haemorrhage (6.6% vs. 8.7%, *p* = 0.004) and gastrointestinal bleeding (2% vs. 2.4%, *p* < 0.0001) [69]. Several additional large cohort studies and a meta-analysis similarly support the use of DOACs in patients with cirrhosis and atrial fibrillation, demonstrating lower rates of stroke (pooled HR of 0.58, 95% CI: 0.35–0.96) without a significant increase in overall bleeding compared with warfarin (pooled HR of 1.45, 95% CI: 0.96–2.17) [70]. Two cohort studies examining bleeding risk between apixaban and rivaroxaban in AF have demonstrated lower rates of major haemorrhage and gastrointestinal haemorrhage with apixaban compared with rivaroxaban, in patients with liver disease, but not in those with cirrhosis [65,71].

## 8. Thrombosis-Specific Management

### 8.1. Pulmonary Embolism and Deep Venous Thrombosis

There is limited data examining the optimal treatment of PE/DVT in patients with cirrhosis. Evidence is further limited in those with concurrent thrombocytopenia. Guidelines generally support the same anticoagulation management in compensated liver disease as in the general population [21]. Patients with isolated distal DVTs may be considered for observation and serial imaging if there are other contraindications to anticoagulation. However, progression of the thrombus should support initiation of anticoagulation with evidence to support the use of LMWH in this setting, particularly in those with Child Pugh C cirrhosis. Vitamin K antagonists may also be used if the baseline INR/PT is not prolonged. Alternatively, in compensated cirrhosis, DOACs may be used [21].

Studies specifically examining DOAC use in the treatment of thromboembolism remain limited. Caution is warranted when extrapolating DOAC data from portal or mesenteric vein thrombosis (PVT/SMV) to systemic venous thromboembolism (DVT/PE). In PVT/SMV, anticoagulation is frequently aimed at achieving recanalisation and reducing portal venous pressure, which, in turn, may lower the risk of variceal bleeding and hepatic decompensation. These therapeutic goals are not directly comparable to those with a DVT/PE [68].

A large retrospective cohort study of over 8000 patients with cirrhosis treated for acute VTE compared outcomes between DOACs and warfarin. This cohort included 2361 DOAC-warfarin, 895 apixaban-warfarin, 2161 rivaroxaban-warfarin, and 895 apixaban-rivaroxaban matched pairs. The primary composite outcome, recurrent VTE and major bleeding, was significantly lower with DOACs (HR, 0.72; 95% CI, 0.61–0.85). Notably, apixaban had lower rates of hospitalisation for recurrent VTE compared with warfarin (HR, 0.47; 95% CI, 0.26–0.86), though major bleeding rates were similar with DOACs as a class vs. warfarin (HR, 0.69; 95% CI, 0.57–0.84), consistent with other studies [65].

Current gastroenterology guidelines recommend DOACS can be used for the treatment of DVT/PE, though they do not recommend use in patients with Child-Pugh C cirrhosis. These societies acknowledge the limited data in this area and advise that further studies are required [42,47].

### 8.2. Portal Venous Thrombosis/Superior Mesenteric Vein Thrombosis

There have been several studies examining the use of LMWH and warfarin in cirrhotic patients with PVT/SMV thrombosis and concurrent thrombocytopenia. A meta-analysis of over 500 patients examined both LMWH and warfarin (41%) for portal venous thrombosis with the primary outcome of all-cause mortality. Patients in the anticoagulation and non-anticoagulation groups had similar platelet counts (median 88.5 [IQR 54.0, 149.0] vs. 86.5 [IQR 57.0, 138.0], *p* = 0.880). All-cause mortality was lower in those who were anticoagulated (adjusted sub-distribution hazard ratio 0.59; 95% CI: 0.49–0.70), as well as having higher rates of recanalisation (adjusted odds ratio 3.45; 95% CI: 2.22–5.36) [72]. Similarly, a meta-analysis of over 350 patients on warfarin vs. LMWH vs. no anticoagulation found both forms of anticoagulation effective in treating PVT progression, though LMWH had higher rates of thrombus resolution. Both anticoagulants had lower rates of variceal bleeding than those not receiving anticoagulation. There was no data on platelet levels in this meta-analysis [73]. Both meta-analyses largely included retrospective data and thus must be interpreted with caution.

DOACS are increasingly used to treat PVT in patients with cirrhosis, though the data are more limited than LMWH/UFH [29]. A recent meta-analysis (10 observational studies and 1 randomised controlled trial) of over 550 patients examined PVT recanalisation rates in patients treated with DOACS vs. VKA. Secondary outcomes included PVT progression, major bleeding, variceal bleeding and death. DOACS had a higher pooled rate of PVT recanalisation (Relative risk (RR) = 1.67, 95% CI: 1.02–2.74, I^2^ = 79%) and a lower pooled risk of PVT progression (RR = 0.14, 95% CI: 0.03–0.57, I^2^ = 0%). Major bleeding, variceal bleeding and death were similar between the different treatment groups [74]. A further study of 94 patients with compensated cirrhosis and acute portal vein thrombosis evaluated the efficacy and safety of rivaroxaban compared with dabigatran. Complete or partial recanalisation was achieved in 75% of patients treated with rivaroxaban (39/52) and 79% of those treated with dabigatran (33/42). The rivaroxaban group had a higher rate of complete recanalisation than the dabigatran group, though this difference was not statistically significant (46 vs. 40%, *p* = 0.581). There were no significant differences in major or minor bleeding events between the rivaroxaban and dabigatran groups (major bleeding: 6% vs. 2%, *p* = 0.646; minor bleeding: 12% vs. 12%, *p* = 0.691) [75].

DOACS may also have benefits in the treatment of more chronic portal venous thrombosis. The definition for a chronic PVT is variable but generally accepted as being present for more than 1–6 months without acute symptoms such as abdominal pain or bowel ischemia [76]. A prospective study evaluated the treatment of chronic portal vein thrombosis in 26 patients receiving rivaroxaban and 14 receiving dabigatran (all Child–Pugh A with platelet counts >50 × 10^9^/L), compared with 40 propensity-matched controls. At 6 months, complete or partial recanalisation was achieved in 28.2% of patients receiving DOACS (11/39) compared with 2.6% in the control group (1/38; *p* < 0.05). There was no significant difference in bleeding rates between the DOAC group (3/39) and the control group (1/38; *p* > 0.05) [77].

Current gastroenterology and haematology guidelines support the use of DOACS for PVT in patients with compensated cirrhosis [29,42].

## 9. Anticoagulation with Cirrhosis-Related Thrombocytopenia

There is extremely limited evidence to guide anticoagulation selection in those with thrombosis and cirrhosis-related thrombocytopenia.

LMWH/UFH and warfarin can be used in both compensated and decompensated cirrhosis [42]. Consensus guidelines, based mainly on expert opinion, recommend a standard LMWH dose if platelets are >50 × 10^9^/L, with reduced dosing for patients with platelet counts between 25 and 50 × 10^9^/L. Intermediate to low-dose LMWH can also be used if there are concerns about bleeding risk [21]. There is limited evidence supporting the use of therapeutic LMWH or unfractionated heparin in patients with platelet counts below 25 × 10^9^/L [49,78]. Warfarin is not recommended in patients with platelet counts below 50 × 10^9^/L due to bleeding risk [49,78].

There is increasing evidence to support the use of DOACS in patients with compensated and even decompensated cirrhosis; however, there is minimal evidence to support DOAC use in the setting of significant thrombocytopenia related to cirrhosis. Haematology guidelines based on retrospective data of the CAT population do not currently recommend their use if the platelet count is <50 × 10^9^/L [48]. Further studies are required to support DOACS and dosing strategies in cirrhosis-related thrombocytopenia.

## 10. Alternatives for Patients with Cirrhosis Unsuitable for Anticoagulation

Inferior vena cava (IVC) filters may be considered in the setting of an acute proximal DVT when anticoagulation is contraindicated, although data on patients with cirrhosis are limited. Removal of the IVC filter is advised at the earliest opportunity to minimise the risk of long-term complications. IVC filters may be used safely in the setting of severe thrombocytopenia and coagulopathy [25,79].

For those with absolute contraindications to anticoagulation, left atrial appendage occlusion can be considered, with extreme caution, to reduce stroke risk in those with atrial fibrillation. However, two large cohort studies have demonstrated that this procedure is associated with significantly higher rates of complications, including increased mortality (OR: 8.6, 95% CI: 4.1–17.9; *p* = < 0.001), intracranial haemorrhage, retroperitoneal haemorrhage, transient ischemic attack, and venous thromboembolism in those with cirrhosis. The authors of this study postulate that this may be due to the need for increased anticoagulation therapy both pre- and post-procedurally [80,81].

## 11. Future Directions

Whilst significant progress and understanding regarding cirrhosis and haemostasis have occurred in recent years, this cohort remains challenging to treat. Patients with advanced liver disease (Child–Pugh C), often accompanied by significant thrombocytopenia, have limited anticoagulation options owing to a paucity of safety data. This is despite Child-Pugh C patients representing the cohort at the highest risk of thromboembolic complications, particularly portal vein thrombosis.

Currently, there are limited risk scores to predict which patients are at the highest risk of bleeding and thrombotic events. In particular, patients with PVT have a variable clinical course, with 5–30% having spontaneous recanalization [10,27]. Functional coagulation testing has recently made significant advances, helping to guide management of patients with cirrhosis, particularly in the setting of haemorrhage. However, the role of VET and global coagulation assays in patients with cirrhosis, thrombosis, and thrombocytopenia remains an area of ongoing research [38]. There remains a lack of consensus on how to integrate emerging biomarkers or global coagulation assays into clinical practice and risk scores. The development of integrated risk prediction models incorporating clinical variables, platelet count, imaging features, biomarkers, and genetic factors would support more individualised anticoagulation strategies in this cohort [82].

Looking forward, several areas of research are critical to inform practice. Future randomised controlled trials and prospective observational studies should ideally be stratified by both Child–Pugh class and platelet count to more accurately define the safety and efficacy of anticoagulation as well as therapeutic thresholds. Such stratification would enable a more nuanced risk-benefit assessment in this population but may not be feasible. Given the challenges of enrolling these patients in randomised controlled trials, large prospective cohort studies, and registry-based analyses may be useful for informing future anticoagulation strategies. Additionally, ongoing pharmacokinetic and pharmacodynamic studies of anticoagulants in cirrhosis are prudent to guide drug selection and dosing. Further research is also required to define the platelet threshold for initiating anticoagulation and to determine how TPO-RA may be incorporated into care.

Despite the perceived risk of anticoagulation in cirrhosis, emerging evidence suggests it may have additive benefits, irrespective of treatment of the underlying thrombosis. A recent meta-analysis demonstrated that anticoagulation in patients with portal venous thrombosis (LMWH or VKA vs. no anticoagulation) could reduce all-cause and liver-related mortality. This was irrespective of thrombosis severity and recanalisation [72]. This may be due to decreased intrahepatic coagulation activation, though further research is required to better understand the underlying mechanisms and role in the setting of co-existent thrombocytopenia [49].

## 12. Conclusions

Cirrhosis-associated thrombocytopenia is an increasingly common clinical problem and frequently coexists with an elevated risk of thromboembolism. This combination makes anticoagulation decisions particularly challenging. Those with moderate thrombocytopenia and compensated cirrhosis have increasing anticoagulation options, including LMWH, VKA and DOACS. However, those with severe thrombocytopenia are largely limited to reduced-dose LMWH (Table 3). Robust data in patients with severe thrombocytopenia and decompensated liver disease is lacking largely due to their exclusion from clinical trials. Prospective or large real-world cohort studies are therefore needed to define safe platelet thresholds for anticoagulation, clarify the role of functional coagulation testing, and better guide antithrombotic therapy in this complex and growing patient population.

## Figures and Tables

**Table 1:** Child–Pugh Classification of Liver Disease Severity [2].

Parameter	1 Point	2 Points	3 Points
Total bilirubin (µmol/L)	<34.2	34.2–51.3	>51.3
Serum albumin (g/L)	>35	28–35	<28
INR	<1.7	1.7–2.3	>2.3
Ascites	Absent	Slight	Moderate
Hepatic encephalopathy	Absent	Grade I–II	Grade III–IV

**Table 2:** Mechanisms of thrombocytopenia in cirrhosis [18].

Decreased platelet production	Reduced hepatic thrombopoietin (TPO) synthesis and impaired c-MPL signalling result in decreased megakaryopoiesis. Bone marrow suppression from alcohol use, chronic hepatitis C infection, and iron overload.
Increased platelet destruction	Reduced ADAMTS13 levels and activity. Immune-mediated platelet clearance. Consumptive thrombocytopenia, particularly during infection or systemic inflammation.
Splenic sequestration	Portal hypertension-associated splenomegaly causes increased pooling and premature clearance of circulating platelets.

**Table 3:** CTCAE Grading of Thrombocytopenia [52].

CTCAE Grade	Platelet Count (×10^9^/L)
Grade 1	<LLN–≥75
Grade 2	50–<75
Grade 3	25–<50
Grade 4	<25

LLN: lower limit of normal.

**Table 4:** Suggested anticoagulation by Child–Pugh class and platelet count (×10^9^/L) [17,24,26,27,30,34,35,36,37].

Child–Pugh A				
Platelet count (×10^9^/L)	<25	25–50	50–100	>100
Warfarin	Avoid	Avoid	Use with caution	Acceptable
LMWH/UFH	Avoid/consider reduced dosing with extreme caution	Use with caution, consider dose reduction	Acceptable	Acceptable
Apixaban	Avoid	Avoid	Use with caution	Acceptable
Rivaroxaban	Avoid	Avoid	Use with caution	Acceptable
Dabigatran	Avoid	Avoid	Use with caution	Acceptable
Child–Pugh B				
Platelet count (×10^9^/L)	<25	25–50	50–100	>100
Warfarin	Avoid	Avoid	Use with caution	Use with caution
LMWH/UFH	Avoid/consider reduced dosing with extreme caution	Use with caution, consider dose reduction	Acceptable	Acceptable
Apixaban	Avoid	Avoid	Use with caution/consider reduced dose	Use with caution/consider reduced dose
Rivaroxaban	Avoid	Avoid	Avoid	Avoid
Dabigatran	Avoid	Avoid	Use with extreme caution/consider reduced dose	Use with extreme caution/consider reduced dose
Child–Pugh C				
Platelet count (×10^9^/L)	<25	25–50	50–100	>100
Warfarin	Avoid	Avoid	Use with caution	Use with caution
LMWH/UFH	Avoid/consider reduced dosing with extreme caution	Use with caution, consider dose reduction	Acceptable	Acceptable
Apixaban	Avoid	Avoid	Avoid	Avoid
Rivaroxaban	Avoid	Avoid	Avoid	Avoid
Dabigatran	Avoid	Avoid	Avoid	Avoid

## Data Availability

No new data were generated or analysed in this study.

## Abbreviations

The following abbreviations are used in this manuscript:

MASLD	Metabolic dysfunction-associated steatotic liver disease
DVT	Deep venous thrombosis
PE	Pulmonary embolism
TPO	Thrombopoietin
vWF	von Willebrand factor
CAT	Cancer-associated thrombosis
LMWH	Low molecular weight heparin
UFH	Unfractionated heparin
DOAC	Direct oral anticoagulants
VKA	Vitamin K antagonists
PVT	Portal venous thrombosis
SMV	Superior mesenteric vein
VTE	Venous thromboembolism
VET	Viscoelastic testing
ROTEM	Rotational Thromboelastometry
TEG	Thromboelastography
